# VITAMIN D LEVELS AND LIPID PROFILE IN PATIENTS UNDERGOING BARIATRIC SURGERY

**DOI:** 10.1590/0102-672020230035e1753

**Published:** 2023-07-28

**Authors:** Isabelle Maria Cabral do Nascimento, Bruna Merten Padilha, Maria Lucia Diniz Araujo, Palena Cabral da Silva, Gisele Almeida de Noronha, Poliana Coelho Cabral, Álvaro Antonio Bandeira Ferraz

**Affiliations:** 1 Universidade Federal de Pernambuco – Recife (PE), Brazil;; 2 Universidade Federal de Alagoas – Maceió (AL), Brazil;; 3 Universidade Católica de Pernambuco – Recife (PE), Brazil;; 4 Centro Universitário UniFBV – Recife (PE), Brazil.

**Keywords:** Bariatric Surgery, Obesity, Vitamin D, Cirurgia Bariátrica, Obesidade, Vitamina D

## Abstract

**BACKGROUND:**

Vitamin, mineral, and metabolic deficiencies occur in the postoperative period of bariatric surgery, in the short and long term, and are worrisome intercurrences.

**AIMS:**

To evaluate the association of serum vitamin D levels with the lipid profile in obese patients undergoing bariatric surgery.

**METHODS:**

Case series of patients assisted from 2010 to 2018, in a private hospital of medium and high complexity, who underwent bariatric surgery using sleeve gastrectomy or Roux-en-Y gastric bypass techniques, monitored by the same surgeon. Sociodemographic, clinical, laboratory, and anthropometric data were collected preoperatively and at 6, 12, and 24 months after surgery.

**RESULTS:**

A total of 156 individuals, mostly female (75.6%) were monitored. The most frequent comorbidities were hepatic steatosis (76.3%) and hypertension (48.27). Regarding preoperative vitamin D levels, only 18.9% of the population had a satisfactory level (≥30 ng/mL). There was a reduction in weight and an improvement in the lipid profile after surgery. Significant correlations were observed between the lipid profile and vitamin D concentration only in the sample submitted to the Roux-en-Y gastric bypass technique: negative correlation between total cholesterol and vitamin D two years after surgery; positive correlation between triglycerides and vitamin D one year after surgery; and negative correlation between high-density lipoprotein and vitamin D two years post-surgery.

**CONCLUSIONS:**

It is essential to routinely monitor vitamin D levels and lipid profile pre- and postoperatively in order to avoid damage associated with this vitamin deficiency.

## RESUMO

**RACIONAL:**

Deficiências vitamínicas, minerais e metabólicas ocorrem no pós-operatório de cirurgia bariátrica, a curto e longo prazo, sendo intercorrências preocupantes.

**OBJETIVOS:**

Avaliar a associação dos níveis séricos de vitamina D com o perfil lipídico, em pacientes obesos submetidos à cirurgia bariátrica.

**MÉTODOS:**

Série de casos de pacientes atendidos de 2010 até 2018, em hospital privado de média e alta complexidade, submetidos à cirurgia bariátrica pelas técnicas da gastrectomia vertical e derivação gástrica em Y de *Roux*, acompanhados pelo mesmo cirurgião. Foram coletados dados sociodemográficos, clínicos, dados laboratoriais e antropométricos no pré-operatório, 6 meses, 12 meses e 24 meses após cirurgia.

**RESULTADOS:**

Foram acompanhados 156 indivíduos, maioria sexo feminino (75,6%), comorbidades mais frequentes foram esteatose hepática (76,3%) e hipertensão (48,27). Em relação aos níveis de vitamina D pré-operatórios, apenas 18,9% da população apresentaram níveis satisfatórios (=30 ng/mL). Observou-se redução do peso e melhora do perfil lipídico pós-cirúrgico. Sobre as correlações entre o perfil lipídico e concentração de vitamina D foram observadas correlações significativas apenas na amostra que passou pela técnica cirúrgica derivação gástrica em Y de Roux: correlação negativa entre o colesterol total e vitamina D após 2 anos de cirurgia; correlação positiva entre triglicerídeo e vitamina D 1 ano pós-operatório; e correlação negativa entre HDL e vitamina D 2 anos pós-operatório.

**CONCLUSÕES:**

é essencial acompanhar os níveis de vitamina D e perfil lipídico no pré e pós-operatório de forma rotineira a fim de evitar danos relacionados a deficiência dessa vitamina.

## INTRODUCTION

Obesity is considered one of the major public health problems today. One in eight adults worldwide is obese. It is estimated that, in 2025, about 700 million individuals will be obese^37^.

Bariatric surgery has become a valuable resource in cases of severe obesity with unsuccessful clinical treatment, reducing mortality and improving clinical comorbidities, especially cardiometabolic diseases and cancer^16^. On the other hand, bariatric surgery is associated with an array of vitamin, mineral, and metabolic deficiencies in the short and long term, and these are intercurrences of concern not only in the preoperative period, but also in the postoperative^10,43^.

Among the deficiencies, hypovitaminosis D is common in obese individuals and can be aggravated after bariatric surgery. Quite often, serum levels of 25-hydroxyvitamin D (25(OH)D) do not rise after surgery, even with supplementation, that is, its insufficiency can put obese individuals at significant risk, and may lead to osteoporosis, osteopenia and fractures, in addition to other diseases^21,46^.

Another independent risk factor for morbidity and mortality is dyslipidemia. This disorder also seems to be related to low levels of vitamin D, which is why the beneficial effects of this vitamin have been suggested in the literature as an ally to reduce comorbidities associated with obesity^7,30,32^.

The objective of this study was to evaluate the association of vitamin D serum levels with the lipid profile in obese patients undergoing bariatric surgery.

## METHODS

This was a case series study, carried out in the general surgery outpatient clinic of a medium and high complexity private hospital in Recife (PE), Brazil. Data collection was conducted by a professional nutritionist, using medical/nutritional follow-up cards.

Data were collected from patients assisted from 2010 to 2018, who underwent bariatric surgery using sleeve gastrectomy (SG) or Roux-en-Y gastric bypass (RYGB) bariatric surgery techniques, monitored by the same surgeon. The SG or simply “sleeve” is a fundamentally restrictive technique that reduces the size of the stomach. RYGB is a mixed technique, with food restriction and malabsorption intended to cause weight loss^10,18^.

Patients included in the study were between 20 and 70 years of age, of both genders, with a body mass index (BMI) between 30 and 60 kg/m², who underwent bariatric surgery (SG or RYGB), who had a complete medical record including sociodemographic, clinical, laboratory and anthropometric data preoperatively and at 6, 12, and 24 months after surgery. Patients with human immunodeficiency virus (HIV) and autoimmune diseases were excluded.

The sociodemographic and clinical variables data collected were: gender, age, duration of surgery, type of surgery performed, and the presence of comorbidities such as systemic arterial hypertension (SAH), diabetes mellitus (DM), dyslipidemia and hepatic steatosis. SAH was defined as systolic blood pressure >140 mmHg or diastolic pressure >90 mmHg^36^; DM when fasting blood glucose >126 mg/dL or glycated hemoglobin = 6.5% or when the patient was using hypoglycemic agents^34^; dyslipidemia when total cholesterol (TC) >190 mg/dL, or low-density lipoprotein (LDL) >100 mg/dL or high-density lipoprotein (HDL) <40 mg/dL; triglycerides (TG) >150 mg/dL^17^; and hepatic steatosis diagnosed through abdominal ultrasonography.

The laboratory tests performed were: vitamin D serum levels, TC, LDL, HDL, and TG. Individuals with 25(OH)D ≤20 ng/mL levels were considered deficient in vitamin D; between 21–29 ng/mL levels were considered insufficient, and ≥30 ng/mL, sufficient^41^.

For anthropometric data, BMI and excess weight loss were calculated. Patients with a BMI between 50–60 kg/m² were classified as super obese according to the American Society of Bariatric Surgery classification^9^.

The rate of excess weight loss (%EWL) is the percentage of weight lost in relation to the excess weight value, which was established based on the patient's pre-surgical weight, ideal weight and weight in the follow-up data after surgery (weight loss [kg]) x 100 / excess weight [kg)). Weight loss was considered successful when there was an elimination of at least 50% of pre-surgical excess weight. The BMI value 24.9 kg/m² was considered the ideal weight, since it is the maximum value within the normal range weight, according to the World Health Organization (WHO)^47^.

The database was created using Microsoft Excel 2010 and the analyses were processed using the Statistical Package for the Social Sciences software (SPSS Inc, Chicago, IL, USA), version 20.0. Results were expressed as relative frequency (%), mean and standard deviation (±). Student's *t* test was used to compare continuous variables between the two surgical techniques. Analysis of variance (ANOVA) for repeated measures, with post hoc Bonferroni test, were used to assess the evolution of the anthropometric and biochemical parameters at different time points of the study. In the description of proportions, the binomial distribution was approximated to the normal distribution, with 95% confidence interval. Correlations between variables were assessed using Pearson's correlation test. Associations between categorical variables were assessed using the chi-square test. All differences were considered significant when p<0.05.

The research project was approved by the Research Ethics Committee of the University Hospital of the Universidade Federal de Pernambuco (PE), resolution No. 466/2012 of the National Health Council under protocol number 22169319.3.0000.8807.

## RESULTS

From 2010 to 2018, a total of 2,537 patients underwent bariatric surgery. Out of this, 208 were found eligible and were included in the study. After 24 postoperative months of follow-up, there were losses in the sample (n=52), totaling 156 individuals. The loss corresponded to 25% of the initial sample, with the main reason being discontinuity of outpatient follow-up. However, from the comparison of demographic and clinical variables of patients who concluded the follow-up and those who were considered as losses, no statistically significant differences were evidenced (Table 1).

**Table 1:** Demographic, clinical and laboratory characteristics of the patients included in the study, from 2010 to 2018, and the losses that occurred during follow-up.

		Follow-up			Losses			p*
		n	%	95%CI	n	%	95%CI	
**Gender**								
	Male	38	24.4	17.3–31.8	7	13.5	6.0–24.4	0.10
	Female	118	75.6	68.2–82.7	45	86.5	75.6–94.0	
**Age**								
	<45 years	96	61.5	53.8–68.9	39	75.0	62.2–85.4	0.08
	≥45 years	60	38.5	31.1–46.2	13	25.0	14.6–37.8	
**Hypertension**								
	Yes	76	48.7	41.2–56.6	20	38.5	26.0–52.0	0.20
	No	80	51.3	43.4–58.8	32	61.5	48.0–74.0	
**Diabetes**								
	Yes	48	30.8	23.7–38.0	9	17.3	8.7–29.0	0.06
	No	108	69.2	62.0–76.3	43	82.7	71.0–91.3	
**Dyslipidemia**								
	Yes	49	31.4	23.9–38.9	16	30.8	19.4–44.0	0.93
	No	107	68.6	61.1–76.1	36	69.2	56.0–80.6	
**Hepatic steatosis**								
	Yes	119	76.3	68.7–82.8	40	76.9	64.3–86.9	0.92
	No	37	23.7	17.2–31.3	12	23.1	13.1–35.7	
**Total cholesterol**								
	Undesirable	85	54.8	47.0–62.5	32	61.5	48.0–74.0	0.40
	Desirable	70	45.2	37.5–53.0	20	38.5	26.0–52.0	
**HDL**								
	Undesirable	46	29.9	23.0–37.4	15	28.8	17.8–42.0	0.89
	Desirable	108	70.1	62.6–77.0	37	71.2	58.0–82.2	
**LDL**								
	Undesirable	49	31.6	24.6–39.2	18	34.6	22.6–48.1	0.69
	Desirable	106	68.4	60.8–75.4	34	65.4	51.9–77.4	
**Triglycerides**								
	Undesirable	62	40.0	32.5–47.8	21	40.4	27.7–53.9	0.96
	Desirable	93	60.0	52.2–67.5	31	59.6	46.1–72.3	
**Vitamin D**								
	Deficient	57	38.5	30.9–46.5	13	26.0	15.2–39.2	0.28
	Insufficient	63	42.6	34.8–50.6	26	52.0	38.3–65.5	
	Sufficient	28	18.9	13.2–25.7	11	22.0	12.1–34.7	

* Pearson's χ² test.

95%CI: 95% confidence interval; BMI: body mass index; HDL: high-density lipoprotein; LDL: low-density lipoprotein.

Table 1 presents the characteristics of the individuals who completed the follow-up, showing that females were prevalent in 75.6%. The most frequent comorbidities were hepatic steatosis (76.3%) and SAH (48.27). Age ranged from 20 to 68 years, with a mean of 40±11.2 years. As for the lipid profile, 54.8% of the sample had undesirable values for TC, 40.0% had undesirable values for TG, 31.6% for the LDL fraction, and 29.9% for HDL. As to vitamin D, only 18.9% of the sample had satisfactory preoperative 25(OH)D levels (≥30 ng/mL).

Table 2 shows the records of weight evolution, BMI, lipid profile and vitamin D, according to the surgery performed. Weight reduction was significant for both patients who underwent SG and RYGB. Multiple comparisons showed differences between all follow-up periods (p<0.05), except between 12 and 24 months in patients undergoing RYGB. BMI also had a significant reduction in the two surgeries performed in all periods, except between 12 and 24 months in SG patients.

**Table 2:** Evolution of weight, body mass index, vitamin D and lipid profile of patients submitted to sleeve gastrectomy or Roux-en-Y gastric bypass, preoperatively and postoperatively at 6, 12 and 24 months.

		Sleeve gastrectomy					Roux-en-Y gastric bypass				
		n	Mean	SD	95%CI	p*	n	Mean	SD	95%CI	p*
**Weight**		60					76				
	Preoperative		104.13^a^	13.71	100.59–107.67	<0.001		116.83^a^	22.38	111.72–121.94	<0.001
	Postoperative 6 months		80.66^b^	11.31	77.73–83.58			89.10^b^	17.07	85.19–93.00	
	Postoperative 12 months		70.27^c^	11.50	67.29–73.24			76.60^c^	15.16	73.13–80.06	
	Postoperative 24 months		71.48^c^	11.75	68.45–74.52			75.22^c^	15.99	71.57–78.87	
**BMI**		60					76				
	Preoperative		39.09^a^	3.50	38.19–40.00	< 0.001		42.28^a^	5.11	41.11–43.44	<0.001
	Postoperative 6 months		30.31^b^	3.39	29.44–31.19			32.39^b^	4.79	31.30–33.49	
	Postoperative 12 months		26.41^c^	3.33	25.55–27.27			27.68^c^	3.87	26.80–28.56	
	Postoperative 24 months		26.83^c^	3.45	25.94–27.72			27.11^c^	3.99	26.20–28.02	
**Total Cholesterol**		52					65				
	Preoperative		201.87^a^	39.12	190.98–212.76	0.011		194.90^a^	39.81	185.04–204.77	<0.001
	Postoperative 6 months		185.69^b^	32.67	176.60–194.79			161.34^b^	33.21	153.11–169.57	
	Postoperative 12 months		192.21^ab^	33.18	182.97–201.45			157.08^b^	30.39	149.54–164.61	
	Postoperative 24 months		197.31^ab^	39.16	186.41–208.21			161.94^b^	31.43	154.15–169.73	
**HDL**		37					61				
	Preoperative		50.37^a^	12.06	46.35–54.39	<0.001		44.88^a^	11.09	42.04–47.72	<0.001
	Postoperative 6 months		50.48^a^	13.21	46.08–54.88			42.04^a^	9.74	39.54–44.53	
	Postoperative 12 months		58.92^b^	14.29	54.16–63.68			49.25^b^	12.41	46.07–52.43	
	Postoperative 24 months		64.37^c^	14.85	59.42–69.33			55.27^c^	12.84	51.98–58.56	
**LDL**		37					61				
	Preoperative		125.87^a^	32.89	114.90–136.83	0.088		117.12^a^	32.31	108.84–125.39	<0.001
	Postoperative 6 months		118.29^a^	28.05	108.94–127.65			97.90^b^	27.80	90.78–105.02	
	Postoperative 12 months		114.54^a^	26.25	105.79–123.30			90.16b.c	25.06	83.74–96.58	
	Postoperative 24 months		111.91^a^	39.66	98.69–125.14			89.93^c^	24.36	83.69–96.17	
**Triglycerides**		52					64				
	Preoperative		160.77^a^	86.12	136.79–184.75	<0.001		167.07^a^	80.19	147.04–187.10	<0.001
	Postoperative 6 months		100.31^b^	34.15	90.80–109.82			104.16^b^	51.89	91.19–117.12	
	Postoperative 12 months		90.79^c^	35.78	80.83–100.75			83.44^c^	32.58	75.30–91.57	
	Postoperative 24 months		99.58b,c	62.74	82.11–117.04			87.05^c^	35.88	78.08–96.01	
**Vitamin D**		33					40				
	Preoperative		21.82^a^	6.70	19.44–24.19	<0.001		23.26^a^	6.74	21.11–25.42	0.073
	Postoperative 6 months		27.67^b^	8.96	24.49–30.85			25.26^a^	8.08	22.68–27.85	
	Postoperative 12 months		27.56^b^	7.33	24.96–30.16			26.11^a^	8.06	23.53–28.69	
	Postoperative 24 months		26.81^b^	8.49	23.80–29.82			26.35^a^	8.68	23.58–29.13	

* ANOVA.

BMI: body mass index; HDL: high density lipoprotein; LDL: low-density lipoprotein; 95%CI: 95% confidence interval.

a,b,c indicate significant differences (p<0.05) between the means by Bonferroni's post hoc test.

With regard to TC, a reduction was recorded after SG and RYGB (p<0.001), except after 24 months in SG patients. An increase in the HDL fraction was observed after both surgeries (p<0.001). Multiple comparisons showed that among the patients submitted to SG, there was no difference between the preoperative period and after 6 months, and between 6, 12 and 24 months after the surgery (p<0.001). However, in RYGB patients, there was a significant and progressive increase in the HDL fraction in all the postoperative periods.

A significant reduction in LDL levels was observed in patients undergoing RYGB (p<0.001), but not in those undergoing SG. Multiple comparisons showed that among patients undergoing SG, there was a difference between the preoperative period and 12 months postoperatively and between the preoperative period and 24 months postoperatively. On the other hand, a significant and progressive reduction of LDL was recorded between all-time points (p<0.01), except between 12 and 24 months, in patients undergoing RYGB.

A reduction in TG was recorded in patients undergoing SG and RYGB (p<0.001). Multiple comparisons showed significant differences between all periods (p<0.05), except between 12 and 24 months in both SG and RYGB patients. Vitamin D levels increased in SG patients (p<0.001), but not in those RYGB.

Table 3 shows the correlations between the lipid profile and vitamin D concentration in the four periods studied. Significant correlations were observed in RYGB patients: negative correlation between TC and vitamin D after 24 months; positive correlation between TG and vitamin D after 12 months; and negative correlation between HDL and vitamin D after 24 months postoperatively.

**Table 3:** Correlation between the lipid profile and vitamin D of patients undergoing sleeve gastrectomy and Roux-en-Y gastric bypass preoperatively and postoperatively at 6, 12 and 24 months.

Lipid profile	Time	Preoperative	Post-operative 6 months	Post-operative 12 months	Post-operative 24 months
		Vitamin D			
TC	SG	−0,15	−0,10	−0,12	−0,19
	RYGB	0,09	−0,20	0,03	**-0,31** *
TG	SG	−0,12	−0,19	−0,06	−0,13
	RYGB	−0,19	0,01	**0,24** *	0,01
HDL	SG	−0,09	0,09	−0,03	0,07
	RYGB	−0,16	−0,11	−0,07	**-0,32** *
LDL	SG	−0,10	−0,11	−0,14	0,03
	RYGB	−0,04	−0,16	0,03	−0,24

* Spearman's Rho coefficients (p<0.05).

TC: total cholesterol; TG: triglycerides; HDL: high density lipoprotein; LDL: low density lipoprotein. SG: sleeve gastrectomy; RYGB: Roux-en-Y gastric bypass.

## DISCUSSION

Bariatric surgery is an effective procedure in the treatment of obesity whether or not associated with other comorbidities. This surgery is commonly performed in Brazil, which ranks 2^nd^ in the number of bariatric surgeries performed worldwide^45^.

Most patients who undergo this type of intervention are female, which was also observed in this study, and can be explained by the increasing prevalence of overweight in this population, in addition to the social pressure for a slim body that affects women more intensely^39^.

Individuals with morbid obesity demonstrate changes in lipoproteins, contributing to cardiovascular risk^8,20,23^. According to a study by Bays, et al.^4^, 60% of obese individuals have dyslipidemia and are candidates for surgical treatment. In our study, 31.3% of the patients had dyslipidemia, and undesirable LDL cholesterol fraction was present in more than half of the patients (56.5%).

As regard the surgical techniques employed, SG stood out in this investigation as the most performed procedure, confirming that it is currently the first choice^3,6,15^. SG was the most popular restrictive procedure, in which the loss of excess weight reached 60% in two years. The RYGB reached the excess weight loss of 67–70% after approximately one year; however, it has higher complication rates, both in the immediate and late postoperative period^25,40^.

Bariatric surgery is essential in improving anthropometric and metabolic parameters, such as the lipid profile^2^. In the present study, the lipid profile showed a significant improvement; however, comparing the type of surgery, patients undergoing RYGB had better levels of total cholesterol, LDL, HDL and TG two years after surgery. On the other hand, patients submitted to SG showed only improvement in total cholesterol, in HDL and TG levels. These results are similar to those obtained by Lira et al.^35^ who evaluated 334 SG patients and 178 RYGB; they observed improvement in the lipid profile (TC, LDL, HDL, and TG) after two years with the use of either techniques, but results were more significant in those patients who underwent RYGB. Buchwald et al.^5^ carried out a meta-analysis of 22,000 patients and concluded that 70% showed improvements in their lipoprotein profile after RYGB.

TC decreased between the preoperative period and all follow-up time points, except between 12 and 24 months, when it presented a significant increase in this period due to weight regain. The study by Santos et al.^42^ reported that weight regain in this population occurs after 18 months postoperatively, and may alter the metabolic profile.

In our investigation, we found a significant reduction in LDL, but only in RYGB patients. This is in agreement with the study by Lira et al.^37^ in which the RYGB technique results showed a significant improvement in LDL two years after surgery, when compared with the SG technique.

Mean preoperative HDL levels in this study were significantly lower than the postoperative means and differed between 6 and 12 months and between 12 and 24 months postoperatively. Similar results were observed in the study by Heffron et al.^27^, who recorded a statistically significant increase in mean HDL cholesterol one year after surgery.

The TG mean level exhibited a significant decrease before surgery compared to the postoperative period, and especially considering 24 months after surgery. Lira et al.^35^ showed a reduction in serum TG levels in 40% of patients after 24 months of postoperative follow-up. In addition, the study by Griffo et al.^26^ showed a reduction in TG after two years in RYGB patients.

In regards to bariatric surgery, other methods can be used to improve the lipid profile; and one of them is the control of vitamin D levels. It is known that vitamin D deficiency is a common disorder in the general population, occurring in 30 to 50% of individuals in all age groups. Patients undergoing bariatric surgery usually exhibit hypovitaminosis D before and after surgery. Several studies show prevalence of vitamin D deficiency in pre-bariatric patients^11–14,19,22,24,38^.

In our study, 81.5% of participants had low levels of 25(OH)D (<30ng/m) preoperatively. Similar frequencies were found in the literature retrieved. De Luis et al.^14^ studying 115 women applicants for bariatric surgery, found a frequency of 71.3% in 25(OH)D levels <30ng/mL. Aridi et al.^1^ reported hypovitaminosis D in 91.5% of 257 patients eligible for bariatric surgery, and Santos et al.^42^ reported that 79.1% of patients had low vitamin D preoperatively.

Low concentrations of 25(OH)D are associated with an unfavorable lipid profile^31^. In our study, there was a significant and negative relationship between TC and vitamin D two years after surgery in RYGB patients. Chaudhuri et al.^7^ described increased TC levels associated with 25(OH)D deficiency in 150 Indian individuals not undergoing bariatric surgery.

TG was positively correlated with vitamin D one year after RYGB. There are reports in the literature of positive associations between serum 25(OH)D and TG^29,44^. Researchers concluded that vitamin D supplementation significantly increased TG. This relation can be explained by the extraction of calcium from the intestine, which reduces the calcium in the intestinal lumen, leading to a decrease in the formation of fatty calcium soaps, greater absorption of fatty acids, and increased circulating TG.

In our study, a negative correlation was observed between the HDL serum level and vitamin D after 24 months postoperatively in RYGB patients. Two studies^28,33^ found a negative association between 25(OH)D and HDL, but the results were not significant.

## CONCLUSIONS

It is essential to routinely monitor vitamin D levels and lipid profiles in the pre- and postoperative period of bariatric surgery in order to avoid complications associated with the deficiency of this vitamin. It is widely recognized that obesity and dyslipidemia are associated with an increased risk of cardiovascular disease and improving lipid concentrations can reduce this risk. After bariatric surgery, continuous follow-up is important and necessary individually, for an accurate prescription of vitamin-mineral supplements so as to avoid and/or correct potential nutritional deficiencies, such as the hypovitaminosis D.
